# Evidence-Based Clinical Efficacy of Leukocyte and Platelet-Rich Fibrin in Maxillary Sinus Floor Lift, Graft and Surgical Augmentation Procedures

**DOI:** 10.3389/fsurg.2020.537138

**Published:** 2020-11-24

**Authors:** Mohamadamin Damsaz, Consuelo Zumarán Castagnoli, Majid Eshghpour, Daryouosh Hamidi Alamdari, Aida Hamidi Alamdari, Ziad Eva Fouad Noujeim, Ziyad Samir Haidar

**Affiliations:** ^1^Dentistry Student Research Committee, Mashhad Dental School, Mashhad University of Medical Sciences, Mashhad, Iran; ^2^BioMAT'X R&D&I Laboratory, Universidad de los Andes, Santiago, Chile; ^3^Oral and Maxillofacial Surgery Department, Mashhad Dental School, Mashhad University of Medical Sciences, Mashhad, Iran; ^4^Surgical Oncology Research Center, Emam Reza Hospital, School of Medicine, Mashhad University of Medical Sciences, Mashhad, Iran; ^5^Department of Oral and MaxilloFacial Surgery, Faculty of Dental Medicine, Lebanese University, Beirut, Lebanon; ^6^Programa de Doctorado en BioMedicina, Facultad de Medicina, Universidad de los Andes, Santiago, Chile; ^7^Centro de Investigación e Innovación Biomédica (CIIB), Universidad de los Andes, Santiago, Chile; ^8^Facultad de Odontología, Universidad de los Andes, Santiago, Chile

**Keywords:** maxillary sinus lift, augmentation, schneiderian membrane, platelet concentrates, bone grafting

## Abstract

Bone augmentation techniques have increasingly been indicated for re-creating adequate bone height and volume suitable for dental implant sites. This is particularly applicable in the severely atrophic posterior maxilla where sinus perforation (ruptured Schneiderian membrane) is a very common complication and sinus floor elevation or lift is frequently considered a standard procedure. The augmentation of the maxillary sinus can be performed with or without grafting biomaterials. Herein, numerous biomaterials and bone substitutes have been proposed, primarily to sustain the lifted space. In addition, cytokines and growth factors have been used to stimulate angiogenesis, enhance bone formation as well as improve healing and recovery period, either as the sole filling material or in combination with bone substitute materials. Within such, is the family of autologous blood extracts, so-called platelet concentrates, which are simply the “product” resulting from the simple centrifugation of collected whole blood samples of the patient, immediately pre-surgery. Platelet-Rich Fibrin (PRF), a sub-family of platelet concentrates, is a three-dimensional (3-D) autogenous biomaterial obtained, without including anti-coagulants, bovine thrombin, additives, or any gelifying agents during the centrifugation process. Today, it is safe to say that, in implant dentistry and oral and maxillofacial surgery, PRFs (particularly, the pure platelet-rich fibrin or P-PRF and leukocyte and platelet-rich fibrin or L-PRF sub-classes) are receiving the most attention, essentially due to their simplicity, rapidness, user-friendliness/malleability, and cost-effectiveness. Whether used as the sole “bioactive” filling/additive material or combined with bone substitutes, the revolutionary second-generation PRFs have been very often associated with *promising* clinical results. Hence, this review aims to provide a 10-years update on the clinical effectiveness of L-PRF when applied/used as the “sole” biomaterial in maxillary sinus augmentation procedures. An electronic search using specific keywords for L-PRF and maxillary sinus augmentation was conducted in three main databases (PubMed-MEDLINE database, Google Scholar and Cochrane library) for the period between January 2009–February 2020. The quest yielded a total of 468 articles. Based on the pre-established *strict* inclusion/exclusion criteria, only seven articles were deemed eligible and included in the analysis. Surprisingly, of the 5 studies which used de-proteinized bovine bone mineral (DBBM) in combination with L-PRF, 60% acclaimed no significant effects and only 40% declared positive effects. Of the two articles which had used allogenous bone graft, 50% declared no significant effects and 50% acclaimed positive effects. Only one study had used L-PRF as the sole grafting material and reported a positive effect. Likewise, positive effects were reported in one other study using L-PRF in combination with a collagen membrane. Due to the heterogeneity of the included studies, this review is limited by the inability to perform a proper systematic meta-analysis. Overall, most of the published studies reported *impressive* results of L-PRF application as a grafting material (sole or adjuvant) in maxillary sinus augmentation and dental implant restorative procedures. Yet, distinct technical processing for L-PRF preparation was noted. Hence, studies should be approached with caution. Here in, in sinus lift and treatment of Schneider membrane, the formation of mature bone remains inconclusive. More studies are eagerly awaited in order to prove the beneficial or detrimental effects of PRFs, in general and L-PRFs, in specific; especially in their tissue regenerative potential pertaining to the promotion of angiogenesis, enhancing of cell proliferation, stimulation of cell migration and autocrine/paracrine secretion of growth factors, as well as to reach a consensus or a conclusive and distinct determination of the effect of leukocytes (and their inclusion) on inflammation or edema and pain; a call for standardization in PRFs and L-PRFs composition reporting and regimenting the preparation protocols.

## Introduction

**Figure 1:**
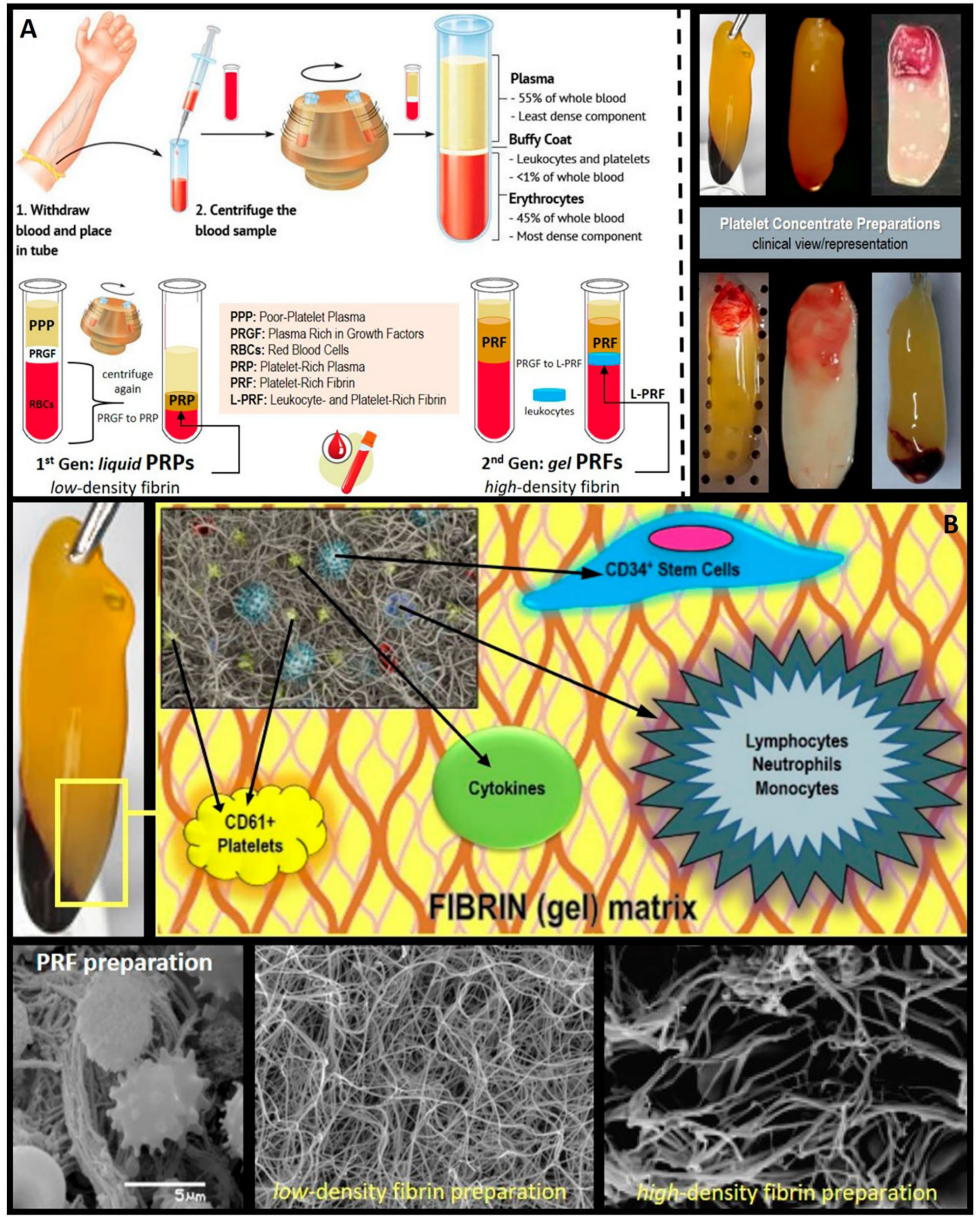
Clinical and histological presentation of L-PRF. **(A)** Platelet concentrates' clinical preparation, types/classes, and clinical illustration/presentation of several platelet-rich fibrin (PRF) and leukocyte and platelet-rich fibrin (L-PRF) preparations (membranes). **(B)** PRF composition/architecture illustration. Schematic representation of PRF bio-components and SEM (scanning electron microscope) micrographs of the PRF membranes displaying its polymerized interconnected fibrin network and large living cell population content.

Albeit the momentous progresses in tissue and defect restoration, regeneration, repair and/or replacement approaches and techniques (and supplies/tools) over the last decades, the posterior maxilla continues to represent a unique and challenging site for dental implant insertion, osseointegration, survival and success, mainly due to its often poor bone quality and deficient bone volume as a result of ridge resorption, atrophy, and sinus pneumatization. While the reconstruction of the posterior maxillary bone volume can be achieved by different procedures such as Le Fort I osteotomies, onlay or inter-positional bone grafts, sinus lifts and augmentation is still considered one of the most predictable regimens. Indeed, bone augmentation techniques have increasingly been indicated for re-creating adequate bone height, volume and density suitable for dental implant sites, particularly applicable in the *severely* atrophic posterior maxilla where sinus perforation is a very common complication and sinus floor elevation or lift is frequently considered a standard procedure. Undeniably, a healthy (and un-ruptured) Schneiderian membrane is deemed “essential” for the successful integration of *any* grafting materials into the maxillary sinus and subsequently, the high survival rates for implants inserted into “augmented” sites. Here in, numerous biomaterials and bone substitutes have been proposed for application in the restoration and reconstruction of posterior maxillary bone volume and maxillary sinus floor lift procedures, mainly to sustain the lifted space. Those include (yet not limited to) autogenous/autograft, freeze-dried bone allograft, xenograft, alloplastic bone (1–3), and, recently, a noteworthy increase in the clinical application of autologous blood extracts, i.e., platelet concentrates (Figure 1A), Briefly, platelet concentrates are growth factor-rich products derived/obtained *via* the simple and rapid (chair-side) centrifugation of collected autologous whole blood from the patient. Such “bioactive additives” have been thus far used in sinus augmentation either as the sole filling material or in combination with other bone substitute materials (4–8). In clinically-usable preparations (surgical adjuvants), the preparation procedure may enhance, accelerate, and promote tissue (soft and hard) wound healing and regeneration (guided bone regeneration) due to the potential to allow the gathering and concentration of platelets and other therapeutic blood constituents (fibrinogen/fibrin, growth factors, leukocytes, and circulating cells) *in situ*. Hence, platelet concentrates do comprise bioactive properties essential for promoting the healing process(es) and period (9, 10), and remodeling of bone grafts and protection (11–13). This have led to the noted interest and increase in their application as a novel therapeutic adjuvant in dentistry and oral surgery (8, 14–22). However, despite the promising clinical observations, their overall effectiveness remains debated today. The main reasons include: contradictory clinical outcomes, insufficient high-quality evidence-based literature, and poor identification of the characteristics of the end-products (and preparation protocols) used in research studies; along with—until recently—a lack of proper nomenclature to typify these concentrates (23–27). In fact, in 2009, the first “classification” consensus (7) was published, categorizing four particular platelet concentrate sub-families relying on differences in biological components (fibrin and cell), properties (gelification), and possible applications: pure platelet-rich plasma (P-PRP), leukocyte and platelet-rich plasma (L-PRP), pure platelet-rich fibrin (P-PRF), and leukocyte and platelet-rich fibrin (L-PRF). Nowadays, when compared to the PRPs, it can be stated that in oral and maxillofacial surgery, the PRFs (P-PRF and L-PRF sub-families; not including red blood cells within) have and continue to receive the highest consideration and hype. This is, as we have recently studied (27–29), is mainly due to (i) *pre-op* simplicity and rapidness of biomaterial attainment and preparation; (ii) *intra-op* user-friendliness in terms of handling and overall malleability; and (iii) *post-op* prognosis in terms of clinical results, pain, edema and cost-effectiveness (5, 7, 23–27).

PRF and L-PRF are second-generation “autologous” platelet concentrates of collected whole venous blood from the patient immediately pre-op (10, 12). Briefly, can be described as a fibrin gel-like material, that is polymerized slowly and strongly abundant in growth factors, platelets, leukocytes (almost half of the initial blood harvest), and lymphocytes is collected, following the simple and rapid (~10 min) centrifugation (*please note that preparation protocols vary*) of blood (10 mL), in vacutainer tubes, without anti-coagulant, bovine thrombin, additives or any other gelifying agent (i.e., *under naturally physiological concentrations of autologous thrombin*) (8, 27–30). The resulting and/or gathered “clot” (now, bioactive material) is stable, resilient, strong, adhesive, and malleable (Figure 1A), where it can be easily cut and/or adapted into different anatomical defects and applications: blended/mixed with bone grafting material, applied as filling material in a direct way, or even compacted into a stronger fibrin membrane. Alongside this established clinical ease of use and handling, the biochemical composition of the PRF by-products provides it with attractive biochemical properties, hemostatic, angiogenic, osteogenic, anti-inflammatory, anti-microbial, pain-inhibitory, and wound-healing characteristics (5, 27–29, 31), rendering PRFs, at least desirable and revolutionary.

Can be used as an adjuvant therapeutic technique following intraoral surgical procedures to enhance tissue regeneration and wound healing (8, 28, 32), L-PRF, as mentioned earlier, belongs to the second generation of autologous platelet concentrates; where it comprises proteins, cytokines, leukocytes, growth factors, and a stem cell-content (11, 29, 33). L-PRF has a *more* convenient fibrin network (Figure 1B) that is suitable for cell migration with ability and capacity to store (and release or deliver) cytokines and growth factors—when compared to PRP (30). In addition, the presence of leukocytes has been suggested by some to be *highly favorable* due to its strong influence on the healing process as a result of the chemotactic recruitment of cells and control of the inflammatory environment (28, 33–39). Accumulating evidence, to date of this review, shows that L-PRF membranes can actively produce and release abundant concentrations of growth factors and cytokines for up to 28 days post-preparation (29). This phenomenon is achieved via activating the platelets, which in turn secrete significant quantities of essential growth factors and proteins including Vascular Endothelial Growth Factor (VEGF), Transforming Growth Factor-β1 (TGF-β1), Bone Morphogenetic Proteins (BMPs), Transforming Growth Factor-β2 (TGF-β2),-which is a major factor in bone healing-, Insulin–like Growth Factor (IGF), and Platelet-Derived Growth factor (PDGF) (11, 19, 31, 33, 40–54).

**Figure 2:**
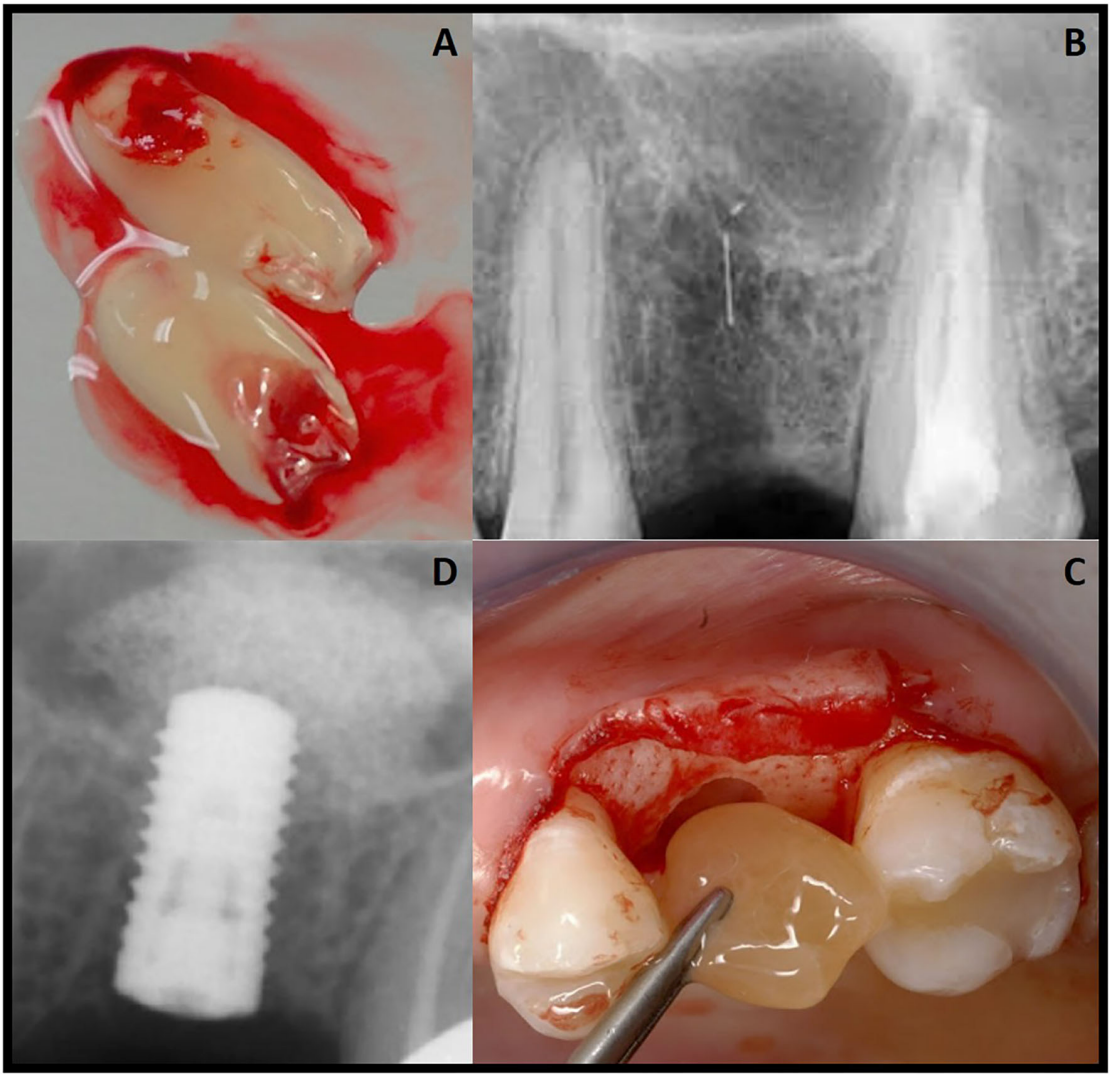
Clinical case presentation of **(A)** prepared L-PRF use/ application in **(B)** a maxillary sinus floor lift and augmentation procedure prior **(C)** to immediate dental implant placement **(D)**.

It is noteworthy here in, to the interested clinical reader, that to obtain a practical or usable L-PRF clot (fibrin), the blood sample should be promptly centrifuged (30, 33, 55). This is because initially, a high concentration of fibrinogen is in the mid-upper part of the tube and, subsequently, there is a transformation of thrombin, which turns into fibrin by centrifugation. In the lower part of the tube there are erythrocytes, and in the middle a resilient, strong and malleable clot of fibrin and at the top there is cellular plasma (27, 56). The resulting clot can be easily cut and/or adapted into the maxillary sinus (Figure 2). It is so vital herein to re-emphasize that the key of the technique is a fast/rapid preparation.

Recently, the clinical effects of L-PRF use and application in maxillary sinus lift procedures have been receiving much attention and to date, no consensus has been reached. While some studies report positive effects, other studies have shown limitations to the clinical efficacy and/or potential of L-PRF for bone formation or that it should be combined with other materials (1) to achieve the desired effect. Hence, such variability of “evidence” alongside the detected confusion, ignited this clinical review in an attempt to carefully evaluate the reported beneficial (or not) effect of L-PRF use during maxillary sinus lift procedures and compare to the most common graft materials used for this particular surgery.

## Methods

The present review was designed and conducted according to the preferred reporting items of the PRISMA (Preferred Reporting Items Systematic review and Meta-Analyses) statement (57, 58).

### Focused PICO Structured Questions

The following PICO was established to formulate the questions:

The population (**P**): comprised of patients in need of maxillary sinus floor augmentation for implant placement; the intervention (**I**) was the exclusive use of L-PRF; the comparison (**C**) was no addition of L-PRF or in combination with other materials, and outcomes (**O**) were bone regeneration. The question was: is the addition of leukocyte platelet rich fibrin (L-PRF)—as a sole grafting material—as efficient as other biomaterials or the combination of both in clinical (human) cases of maxillary sinus floor augmentation procedure?

### Eligibility Criteria

The inclusion criteria were clinical studies of patients who underwent maxillary sinus lift surgery and in which the comparison was between the use of L-PRF alone or in combination with other biomaterials in comparison with standard technics. Different protocols in preparation of L-PRF were included.

The exclusion criteria comprised the use of other platelet concentrates and biological enhancers such as: platelet rich fibrin (PRF), platelet rich plasma (PRP), bone morphogenetic proteins (BMPs) fibrin glue, plasma rich in growth factors (PRGF), enamel matrix derivative (EMD) and recombinant human PDGF (*rh*-PDGF), *in vitro* and animal *in vivo* studies.

### Search Strategy

An electronic search was conducted using three main databases PubMed-MEDLINE database, Google Scholar and Cochrane library/Cochrane Central Register for Controlled Trials) for the period between January 2009 – February 2020. Only studies in English language were included. The last search was performed on the 3rd of February of 2020. The main key words were: “L-PRF” and “maxillary sinus augmentation,” as follows: (a) (“leukocyte and platelet-rich fibrin”(All Fields) AND “sinus” (All Fields)); (b) [ “sinus floor augmentation” (Mesh) OR “sinus lift” (All Fields)] AND [ “L-PRF” (Mesh)].

### Study Selection

Titles and abstracts obtained by the electronic search were screened by four authors. Upon screening, full-text versions of all the eligible articles were obtained and carefully investigated. All authors had to agree on the inclusion criteria, exclusion criteria and the finally selected articles. Any case of disagreement(s) was resolved by an open discussion between the authors and supervised by the corresponding author. No registered incidents of (no) consensus or disagreements between the authors.

### Data Collection Process

Tabulated data extraction included the first author, year of publication, and study design; characteristics of populations; L-PRF preparation procedure, comparison/control; treatment outcomes, complications, and patient-reported outcomes. Five authors triple-checked the tabulated data/item collection process.

### Quality Assessment and Risk of Bias

The quality assessment of all included articles was conducted by four authors in addition to the corresponding author, based on Cochrane Collaboration's tool for assessing risk of bias (59); as is presented in Table 1, classified as: adequate (+), inadequate (-), unclear (^*^) or not applicable (NA); according to the following criteria: (a) randomization (b) allocation concealment (c) blinding of participants personnel and outcome assessors (d) completeness of follow-up (e) selective reporting (statistical reporting) and (f) other bias. Hence, a low risk of bias could be indicated if all the above criteria were met. A moderate risk of bias is indicated if one or more key domains were unclear and, on the other hand, a high risk of bias is indicated if one or more key domains were not met. The conducted Risk of Bias was not evaluated for obtained articles classified as case reports and case series.

**Table 1:** Qualitative analysis of the included controlled clinical trials (sinus augmentation).

**Randomization**	**Allocation concealment**	**Blinding of participants personnel and outcome assessors**	**Completeness of follow-up**	**Selective reporting (statistical reporting)**	**Other bias**	**References**
+	+	*	+	+	+	(60)
+	+	*	+	+	+	(61, 62)
+	*	*	+	+	+	(63)
+	+	*	+	+	+	(64)

## Results

### Study Selection

**Figure 3:**
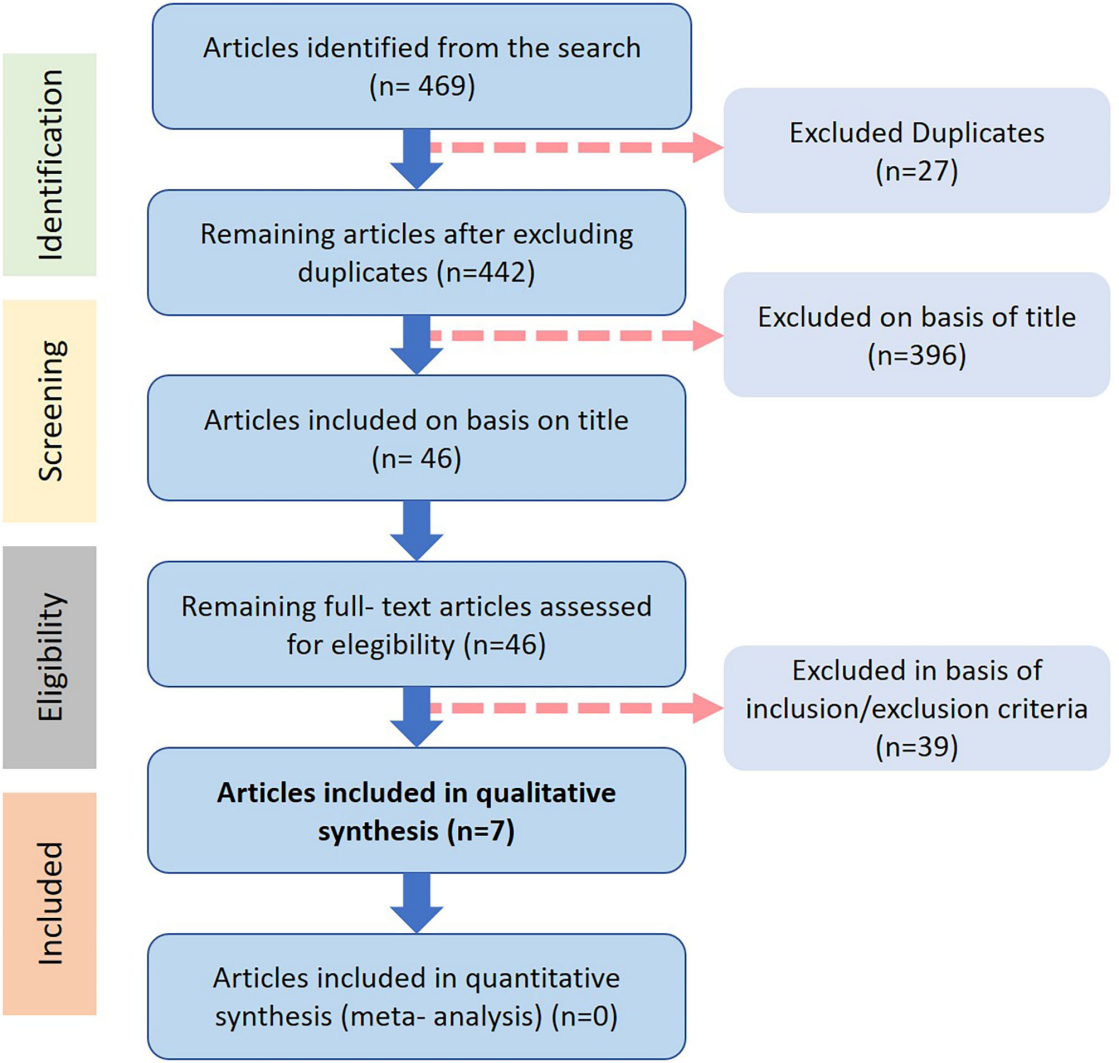
Database search and study selection diagram.

The article selection process is illustrated in Figure 3. The electronic search in PubMed-MEDLINE, Google Scholar and Cochrane Central Register of Controlled Trials (Cochrane Library) respectively provided 469 potentially eligible articles published between the years of 2009 and of 2020, with 27 item duplicates discarded following reading all of the abstracts. Then, 396 articles were excluded on the basis of title and scope, and 46 full-texts were further assessed for eligibility with 39 full-texts then excluded (with reasons) and finally only seven articles met the inclusion criteria and were considered for in-depth comparative analysis. The characteristics of all included studies are presented in Tables 2–4.

**Table 2:** Studies on L-PRF and maxillary sinus augmentation with L-PRF as the SOLE grafting material.

**L-PRF as the SOLE grafting material**						
**Patient characteristics**	**Experimental groups**	**Methodology and recorded parameters**	**L-PRF preparation protocol**	**Surgical intervention**	**Outcome**	**References**
20 patients (8M/12F) AGE: 59.8 ±11.1 years SA: 23	Study 1: L-PRF (Astra Implants) (7) Study 2: L-PRF (Intra-Lock Implants) (13) Control: No.	2–6 years follow up Retro-alveolar and panoramic X-rays. CT scan or low dose volumetric computed radiography (some cases)	400 g 12 min	Classical lateral sinus-lift using the Caldwell-Luc approach.	Bone gain was 8.5–1.2 mm (mean: 10.4 ±1.2 mm). Crestal bone height was very stable. No Implant Lost The use of L-PRF as sole filling material seems to be a reliable surgical option promoting natural bone regeneration	(61)

*AOT, absence of teet; BH, bone height; M, male; F, female; ISQ, Implant stability quotient; NB, new bone; NFB, newly formed bone; NR, not reported; NSC, no statistically comparison; NSSD, no statistically significant difference; RCT, randomized clinical trial; RR, resorption Rate; SA, sinus augmentation; SD, significant difference*.

**Table 3:** Studies on L-PRF and maxillary sinus augmentation with L-PRF in combination with OTHER graft materials.

**L-PRF in combination with OTHER graft materials**						
**Patient characteristics**	**Experimental groups**	**Methodology and recorded parameters**	**L-PRF preparation protocol**	**Surgical intervention**	**Outcome**	**Reference**
1 patient (1M) AGE: 59 years SA: 2	Study: DBBM + L-PRF (3:1) (R) Control: DBBM (L)	Implant stability Augmented bone height CBCT Histology ISQ	3,000 rpm 10 min	Implant placement: 4 months (L-PRF + DBBM) or 8 months (DBBM)	BR: 22.52% (Test) vs 8.95% (Ctrl) ISQ: Above 68 in all implants NFB: 975535 mm^3^ (test) and 2118102 mm^3^ (test), higher with L-PRF Less fibrous tissue in study group The addition of L-PRF might enhance the post-insertion stability of implants during implant healing. L-PRF may accelerate bone healing allowing early placement of dental implants.	(65)
24 patients (14M/10F) AGE: 4 excluded patients (from initial 28) 24 SA	Study: Allogenous freeze dried corticoncellous bone chips + L-PRF (12) Control: Allogenous bone graft (12)	Patients questionnaire with: postop. pain, swelling, sleeping, eating, phonetics, activities of daily living, and missed work days Soft tissue healing (HI)	2,700 rpm 12 min	Lateral window approach	Gradual improvements in postop. pain, swelling, sleeping, eating, phonetics, activities of daily living, and missed work days but no significative Wound healing uneventful HI was higher for test vs. control on days 7 and 14 postop. Better wound healing and patient comfort following direct sinus lifting although the difference did not reach significance.	(60)
10 patients (8M/2F) AGE: 43.5 (study) 46.2 (control) years SA: 11	Study: DBBM + L-PRF (6) Control: DBBM (5)	Radiographic evaluation Histology Histomorpho-metry	300 g 10 min	Lateral wall protocol	NB: 12.95 ± 5.33 (control) vs. 18.35 ± 5.62 (test) (NSSD) Residual bone substitute: 28.54 ± 12.01(control) vs. 19.16 ± 6.89 (test) (NSSD) Bone-to-bone substitute contact (%): 18.57 ± 5.39 (control) vs. 21.45 ± 14.57 (test) (NSSD) Neither advantage nor disadvantage in the application of PRF in combination with DBBM in SA, after 6 months.	(63)
13 patients (9M/4F) AGE: 49.92 ± 10.37 years SA:26	Study: DBBM + L-PRF (13) Control: DBBM (13)	Radiographic residual and augmented bone height	400 g 12 min	Lateral approach Implant surgery after 6 months	NB: 21.25 ±5.59% (control) vs. 21.38 ± 8.78% (test) (NSSD) Soft tissue: 45.96 ± 8.36% (control) vs. 52.67 ± 12.53% (test) (NSSD) Residual bone graft: 32.79 ± 5.89% (control) vs. 25.95 ± 9.54% (test) (NSSD) Bone-to-graft contact: 54.04 ± 8.36% (control) vs. 47.33 ± 12.33% (test) (NSSD) The addition of L-PRF in DBBM did not improve the amount of regenerated bone or the amount of the graft integrated into the newly formed bone under histological and histomorphometric evaluation	(64)
12 patients (6M/6F) AGE: 54.17 ± 6.95 years SA:24	Study: DBBM + L-PRF (12) Control: DBBM (12)	CBCT Histology Histomorpho-metry	300 g 10 min	Lateral window approach Implant placed at 4 (test) or 8 (control) months	NB: 44.58 ± 13.9% (test) vs. 30.02 ± 8.42% (control) Residual graft material: 3.59± 4.22% (test) vs. 13.75 ± 9.99% (control) Soft Tissue: 26.60 ± 11.13% (test) vs. 30.64 ± 12.46% (control) Mean graft volume: 1.68 ± 0.42 cm^3^ (test) vs. 1.46 ± 0.53 cm^3^ (control) (T1)(NSSD) and 1.10 ± 0.25 cm^3^ (test) vs. 0.91 ± 0.35 cm^3^ (SSD) ISQ: 60.90 ± 9.35 (test) vs. 75.13 ± 5.69 (control) (placement) 75.75 ± 6.14 (test) vs. 76.08 ± 5.68 (control) (loading) Adding L-PRF leads to faster bone graft maturation, and this outcome might suggest sinus augmentation with a shorter healing time before implant placement.	(62)

*AOT, absence of teet; BH, bone height; M, male; F, female; ISQ, Implant stability quotient; NB, new bone; NFB, newly formed bone; NR, not reported; NSC, no statistically comparison; NSSD, no statistically significant difference; RCT, randomized clinical trial; RR, resorption Rate, SA, sinus augmentation; SD, significant difference*.

**Table 4:** Studies on L-PRF and maxillary sinus augmentation with L-PRF as an adjuvant in the management of sinus membrane perforation.

**L-PRF as an ADJUVANT in the management of sinus membrane perforation**						
**Patient characteristics**	**Experimental groups**	**Methodology and recorded parameters**	**L-PRF preparation protocol**	**Surgical intervention**	**Outcome**	**References**
1 patient (1M) AGE: 70 years SA: NR AOT:16/17/18	Study: L-PRF as a repair method for sinus membrane perforation (1). Control: No.	8, 14, and 20 months follow up CBCT	3,000 rpm 10 min	Graft material: DBBM Sinus membrane repairing: L-PRF + Collagen	After 6 months grafts ensured none formation form implant installation Bone graft well-delimited to the area of interest No prosthesis failure or periimplantitis The use of L-PRF associated with collagen membrane was efficient for the sealing of the sinus membrane perforation and enabled bone formation for subsequent implant installation.	(66)

*AOT, absence of teet; BH, bone height; M, male; F, female; ISQ, Implant stability quotient; NB, new bone; NFB, newly formed bone; NR, not reported; NSC, no statistically comparison; NSSD, no statistically significant difference; RCT, randomized clinical trial; RR, resorption Rate; SA, sinus augmentation; SD, significant difference*.

### Quality Assessment and Risk of Bias Analysis of Included Studies

The quality assessment of all the included studies in this review is presented in Table 1. Most of the studies were identified with a moderate risk of bias and only one study was recognized with a high risk of bias.

### Results of Individual Studies

Of the seven studies, four used L-PRF in combination with DBBM (63–66), one used L-PRF as a sole grafting material (61) and one utilized L-PRF as a repair material/method for a sinus membrane perforation (62).

The study of Zhang et al. (63) compared the effect of six sinus floors grafted with DBBM and L-PRF with five sinuses grafted with xenograft alone. They described that the newly formed bone in the L-PRF group was 1.4 times higher than that in the control group, without statistical significance. Histologically, a very similar composition and distribution of histologic structures were detected between the groups, with no significant signs of any inflammatory reaction. Evidently, the DBBM particles were distributed homogenously within the augmented area and new bone formation (characterized as woven bone in contrast to the mature skeletal tissue of the alveolar crest, consisting of lamellar bone), was shown to bridge the gaps between the DBBM particles in samples. Furthermore, no significant difference in the percentage of the contour length of the bone substitute material in contact with new bone, was observed, with a bone-to-bone substitute contact of 21.45 ± 14.57% in the DBBM+L-PRF group and 18.57 ± 5.39% in the control group. On the other hand, the study of Pichotano et al. (65), was of split-mouth design in one patient where they compared the combination of xenograft with collagen membrane and L-PRF (experimental side) with DBBM and collagen membrane alone. The magnitude of newly-formed bone was measured using histomorphometric analysis showing a higher proportion in the experimental side (2,118,102 vs. 975,535 mm^3^). Their study described less fibrous tissue within the sinus in the side without L-PRF than the contra-lateral side, at the expense of more newly formed bone. Nizam et al. (64) also used a split-mouth design and compared DBBM + L-PRF (case) and DBBM alone (control). The percentages of newly-formed bone were 21.38 ± 8.78% and 21.25 ± 5.59% for each group, respectively, with no significant differences. They found NSSD in the newly-formed bone ratio, bone graft remnants, fibrous tissue within the sinus and percentage of the bone graft in contact with the newly-formed bone.

Pichotano et al. (66) applied a combination of L-PRF with DBBM for their test group, and DBBM alone for the control group, with the percentages of newly formed bone 44.58 ± 13.9% for the test group and 30.02 ± 8.42% for the control group, showing an increase in new bone formation that favored the L-PRF group, however without statistical significant differences. The team obtained biopsies at 4 months (during implant placement) from the group with bone graft and L-PRF and at 8 months from the control group (xenograft only). Histomorphometric analysis showed increased new bone formation in the experimental group in comparison to the control group (44.58 ± 13.9% and 30.02 ± 8.42%, respectively). No significant differences in the amount of fibrous tissue within the sinus were detected. Additionally, more residual bone graft in the control group reported, yet with no statistical significance.

Simonpieri et al. (61) used L-PRF as a sole grafting material and found that the L-PRF *seemed* to promote bone regeneration according to the results of vertical bone gain: between 8.5 and 12 mm.

### Effect of L-PRF on Soft Tissue Healing

Gurler and Delilbasi (60) reported higher wound healing in the case group (L-PRF + allogenous bone graft) in comparison to the control group (allogenous bone graft alone). The HI scores (healing index) of the case group (4.2 ± 0.9) were higher than the control group (3.6 ± 0.7) on the 7th (4.7 ± 0.4) and 14th post-operative days (4.4 ± 0.5). Therefore, no significant differences were observed (*P* = 0.127 and *P* = 0.189, respectively). The authors also reported “gradual” improvements in post-operative pain, swelling, sleeping, eating, phonetics, activities of daily living, and number of missed working days in the L-PRF group, however no significant differences between the two groups were observed (*P* > 0.05).

## Discussion

This review was designed, aimed and attempted to closely assess the potential benefit (or lack thereof) of L-PRF concentrate and by-product use during maxillary sinus floor/membrane lift, graft and surgical augmentation procedures, not solely as a matrix but also as a bioactive autologous material (62, 67). The included studies were selected on basis of L-PRF/Choukroun's platelet-rich fibrin and not any other type of platelet concentrates such as P-PRF (Pure Platelet-Rich Fibrin), P-PRP (Pure Platelet-Rich Plasma), PRF (Platelet-Rich Fibrin) or PRP (Platelet-Rich Plasma). It should be noted that some reviews mixed up the different types of platelets and considered them the same (59). It also has been reported that the use of PRF in combination with other grafting biomaterials for maxillary sinus floor augmentation seems to provide no *additional* beneficial effects (68). The heterogeneity of platelet concentrates has been explained in many studies (5, 69–73). L-PRF is widely used in many fields such as orthodontics (74), endodontics (75), periodontics (76), skull surgeries (77), aesthetic plastic surgeries (46), and for treatment for Androgenetic Alopecia (78). There are various techniques and protocols to obtain L-PRF based on the rate and time of centrifugation and settings, which do influence the chair-side, histomorphometrical, and clinical outcomes (27, 79). Changes in the time of centrifugation, speed, or even the utilization of different centrifuges can affect the final product in significant ways (27, 80). Therefore, as many researchers agree (81), a clear standardized protocol is much considered necessary.

L-PRF is not just a fibrin membrane but a matrix that contains *all* the *beneficial* substances and cues found in the blood (12). Some studies opted to combine L-PRF with DBBM to enhance osteoconductive properties of the graft and provide a material for a slow rate of absorption (70, 71). This combination may accelerate bone formation and promote wound healing. The mechanism that underlies both events could be the ability of L-PRF to increase blood flow in the sinus cavity via the release of growth factors and moreover its ability to increase osteoblast proliferation *in vitro* stimulating osteo-protegerin secretion in human osteoblasts. Nonetheless, the exact mechanism pathways underlying the role and/or clinical function/effect of L-PRF are not fully understood (72, 73).

It was suggested that L-PRF could be beneficial for bone healing and post-operative discomfort when used in combination with traditional graft materials (29), yet the inclusion of leukocytes have been reported to induce/increase pain and inflammation/edema (82) and accelerate the deterioration of the fibrin (83, 84). Different articles in the research reveal a beneficial effect of L-PRF in maxillary sinus lift procedures, especially when combined with DBBM. Nonetheless in most of the studies, the difference was non-statistically significant. It is noteworthy, that in most, except for Pichotano in 2018 (65) the biopsies were taken at the same time for both groups. Pichotano describes, that maybe the use of L-PRF could accelerate bone formation and therefore enable the insertion of the dental fixture/implant earlier than when using any other protocol. However, this cannot be evidently conclusive and therefore, further longitudinal, randomized, controlled clinical trials are warranted in/to support this assumption.

It is perhaps suitable to re-emphasize to the interested clinical reader that new or modern L-PRF preparations (and protocols), unlike the predecessors, tend to function more as biologically-active biomaterials, scaffolds and/or matrices (platforms) for autologous cells and growth factor release and delivery. Therefore, it is to be considered a “living tissue” preparation for natural guided tissue regeneration rather than just a “growth factor-rich” surgical adjuvant. Yet, its only fair to clarify that this remains an unexplored territory in dental bio-engineering research, to the best of knowledge, and further investigation is encouraged to confirm the reported “clinical observations,” whilst other scientific studies are awaited to accurately characterize and/or elucidate the underlying mechanism(s) of action, from the physical, chemical-biological (histo-morpho-metrical and immune-histo-chemical) and mechanical-rheological aspects of L-PRF, for a more practical understanding, suitable for its use and application (28, 85); a topic currently undergoing vigorous R&D at our BioMAT'X Chile laboratory.

## Conclusion and Closing Remarks

Hundreds of endogenous proteins affect the tissue repair process(s), including angiogenesis, chemotaxis and cell proliferation, with no one exogenic agent effectively governing all these processes. Platelet Concentrates, in general and PRFs, in specific, as a biomedical and biologic tool, aim to quantitively and qualitatively stimulate tissue regeneration via the application/utilization of such autologous proteins and *in situ* enrichment with growth factors. Thus, far, the balanced and gradual release of such bio-active growth factors and cytokines plays a critical role in clinical efficacy and tissue repair time (speed). In summary, our attempted review demonstrates neither an advantage nor a disadvantage, clearly, in the application of PRF preparations and their by-products in combination with bone grafting materials. The use of bone grafts remains much more predictable than the use of L-PRF alone, in particular. The combination of both “biomaterials,” however, could move forward or accelerate healing and the time of dental implant insertion. Hence, in sinus lift and treatment of Schneider membrane, the formation of mature bone remains inconclusive. Therefore, more studies are eagerly awaited utilizing standardized protocols in order to prove the beneficial or detrimental effects of PRFs, in general and L-PRFs, in specific; especially in their tissue regenerative potential pertaining to the promotion of angiogenesis, enhancing of cell proliferation, stimulation of cell migration and autocrine/paracrine secretion of growth factors, as well as to reach a consensus or a conclusive and distinct determination of the effect of leukocytes (and their inclusion) on inflammation/edema and pain.

## Author Contributions

MD: database search, tabulation, and manuscript write-up. CC: database search, tabulation, check, and art-work. ME: database search, tabulation, and manuscript write-up. DA: database search, tabulation, check, quality assessment, and RoB. AA: database search, tabulation, and selected study analysis. ZN: contributed to review idea, database check, discussions, and art-work. ZH: review idea, database check, discussions, manuscript writing, editing, closing remarks, and overall supervision of work. All authors contributed to the article and approved the submitted version.

## Conflict of Interest

The authors declare that the research was conducted in the absence of any commercial or financial relationships that could be construed as a potential conflict of interest.
